# Severe Ketoacidosis (pH ≤ 6.9) in Type 2 Diabetes: More Frequent and Less Ominous Than Previously Thought

**DOI:** 10.1155/2015/134780

**Published:** 2015-06-21

**Authors:** René Rodríguez-Gutiérrez, Carlos R. Cámara-Lemarroy, Dania L. Quintanilla-Flores, Emanuel I. González-Moreno, Juan Manuel González-Chávez, Fernando Javier Lavalle-González, Jose Gerardo González-González, A. Enrique Caballero

**Affiliations:** ^1^Division of Endocrinology, Internal Medicine Department, University Hospital “Dr. José E. González”, Autonomous University of Nuevo León, 64460 Monterrey, NL, Mexico; ^2^Knowledge and Evaluation Research Unit, Division of Endocrinology, Diabetes, Metabolism and Nutrition, Department of Medicine, Mayo Clinic, Rochester, MN 55905, USA; ^3^Department of Internal Medicine, University Hospital “Dr. José E. González”, Autonomous University of Nuevo León, 64460 Monterrey, NL, Mexico; ^4^Latino Diabetes Initiative, Joslin Diabetes Center, Harvard Medical School, Boston, MA 02215, USA

## Abstract

Diabetic ketoacidosis is a life-threatening acute metabolic complication of uncontrolled diabetes. Severe cases of DKA (pH ≤ 7.00, bicarbonate level ≤ 10.0, anion gap > 12, positive ketones, and altered mental status) are commonly encountered in patients with type 1 diabetes and are thought to carry an ominous prognosis. There is not enough information on the clinical course of severely acidotic type 2 diabetes (pH ≤ 6.9) patients with DKA, possibly because this condition is rarely seen in developed countries. In this series, we present 18 patients with type 2 diabetes, DKA, and a pH ≤ 6.9 that presented to a tertiary university hospital over the past 11 years. The objective was to describe their clinical characteristics, the triggering cause, and emphasis on treatment, evolution, and outcomes. The majority of the patients were female (61%). Mean age was 40.66 years (23–59). The patients had been first diagnosed with type 2 diabetes on average 5.27 ± 3.12 years before admission. Glutamic acid decarboxylase (GAD65) antibodies were negative in all patients. The origin of DKA could be attributed to two main causes: treatment omission in 8 (44.4%) patients and infections in 7 (38.8%) patients. The most common symptoms described were general malaise, dyspnea, altered mental status, and abdominal pain. Mean serum glucose on admission was 613.8 ± 114.5 mg/dL. Mean venous pH was 6.84 ± 0.03 with an anion gap of 30.3 ± 2.9 and a venous HCO_3_ level of 3.62 ± 1.35 mmol/L. All patients had acute renal failure on admission, with a mean serum creatinine of 1.57 ± 0.35 mg/dL compared to 0.55 ± 0.21 mg/dL at discharge. All patients received regular insulin infusion, aggressive fluid repletion, and 12 patients (66%) received bicarbonate infusion. Mean total insulin infusion dose was 181.7 ± 90.4 U (on average 0.14 ± 0.05 U/Kg/h). Mean time on infusion was 24.4 ± 12.6 hours. We recorded no mortality in this case series. Mean in-hospital stay was 5.0 ± 4.1 days. In conclusion, very severe DKA in type 2 diabetes is not uncommon in our population, shares many features with non-very-severe cases of DKA (bicarbonate therapy did not make a difference in mortality), and can be managed following standard published or institutional guidelines.

## 1. Introduction

Diabetic ketoacidosis (DKA) is a life-threatening acute metabolic complication of uncontrolled diabetes. This illness results from the relative or absolute deficiency of insulin and an increase in counterregulatory hormones such as glucagon, cortisol, catecholamines, and growth hormone [1, 2]. Despite notable advances in treatment and use of novel drugs with multiple mechanisms of action, hospital admissions due to DKA have increased 30% in the US in the last decade [3]. Classically described in type 1 diabetes, DKA can also occur in type 2 diabetes during catabolic stress scenarios such as infections, surgery, and trauma or late during the natural history of the disease, when the beta-cell function is lost.

Severe cases of DKA (pH ≤ 7.00, bicarbonate level ≤ 10.0, anion gap > 12, positive ketones, and altered mental status) are commonly encountered in patients with type 1 diabetes and are thought to carry an ominous prognosis [2, 4]. The acid-base status in particular has received great attention, due to the potential of bicarbonate-based therapy. There is not enough information on the clinical course of severely acidotic type 2 diabetes patients with DKA, possibly because this condition is rarely seen in developed countries, where there is greater control of diabetes and greater accessibility to medical services. In many developing countries like Mexico, severe DKA in type 2 diabetes patients is very common and represents a therapeutic challenge even for an experienced physician. Despite the fact that several guidelines describe the classification and treatment of severe DKA, most of the recommendations in this scenario are based on case reports and small case series that do not enable the physician to predict the true evolution of this serious illness.

Large case series have been published dealing with the presentation and clinical course of patients with severe DKA, but most of them included either pediatric patients or adults with type 1 diabetes [4–6]. Additionally, the criterion for classifying DKA severity varies widely (pH of <7.2, <7.15, or <7.1, e.g.). Information concerning type 2 diabetes patients with severe acidosis and DKA is quite limited. In this series, we present 18 patients with type 2 diabetes, DKA, and a pH ≤ 6.9 that presented to a tertiary university hospital over the past 11 years, with the objective of describing their clinical characteristics, the triggering cause, and emphasis on treatment, evolution, and outcomes.

## 2. Patients and Methods

Patients were enrolled from the Emergency Department from the University Hospital “Dr. José E. Gonzalez” in Monterrey, Mexico. Cases were identified by electronic and hand search of medical files from January 01, 2002, to November 30, 2013, using the key words “diabetic ketoacidosis,” “hyperglycemic crisis,” “type 2 diabetes,” “acute metabolic diabetes complications,” and related terms. A case was considered when a patient with type 2 diabetes was diagnosed with DKA and the pH was ≤6.9. We arbitrarily defined cases as “very severe DKA” based exclusively on this biochemical variable. In all cases, the chief complaint and the metabolic alterations were attributed to DKA and all alternative diagnoses were ruled out. The files were excluded if the patient had type 1 or type 2 diabetes with a DKA and a pH ≥ 6.91 or hyperglycemic hyperosmolar state. Each case was identified and reviewed to obtain the data by 4 of the authors.

The initial treatment of all patients was carried out in the Emergency Department where vital signs and electrocardiogram were continuously monitored. We extracted patient demographic information, routine biochemical workup, treatment information, and clinical course from patient records in a retrospective manner. In general, institutional treatment guidelines for DKA were followed in all cases, according to published guidelines [1].

## 3. Results

### 3.1. Patients

We identified 18 cases fulfilling our criteria and about the same number were excluded for different reasons. Most patients arrived by emergency services, although four arrived by their own means. Most were of a low socioeconomic and cultural status. Their demographic characteristics are reported in Table 1. The majority of the patients were female (61%). Mean age was 40.66 years (23–59). The patients had been first diagnosed with type 2 diabetes on average 5.27 ± 3.12 years before admission. Seven (38.8%) of the patients have had a previous episode of DKA. Mean body mass index was 22.9 ± 2.9, with a minority of patients being classified as overweight. Five patients (27.7%) were treated with an insulin regimen and 13 (72.3%) used different oral antidiabetic medications before admission. For the patients with insulin therapy, all of them had previously used antidiabetic oral medications on average 3.1 ± 1.2 years. Glutamic acid decarboxylase (GAD65) antibodies were negative in all patients.

### 3.2. Signs and Symptoms

The origin of DKA could be attributed to two main causes: treatment omission in 8 (44.4%) patients and infections in 7 (38.8%) patients [(pneumonia (3), acute pyelonephritis (3), and meningitis (1)]. Classical signs and symptoms of DKA were present in most of our patients. The most common symptoms described were general malaise, dyspnea, altered mental status, and abdominal pain. The majority of patients were clinically dehydrated, with dry mucous membranes. Only two patients were hypotensive on admission (systolic blood pressure < 80 mmHg); mean systolic and diastolic blood pressure on admission were 118.8 ± 21.4 mmHg and 78.3 ± 12.5 mmHg, respectively. Most patients were tachycardic and tachypneic on admission, with a mean heart rate of 98.2 ± 18.0 bpm and a mean respiratory rate of 25.7 ± 4.4 rpm.

### 3.3. Biochemical Variables

Mean serum glucose on admission was 613.8 ± 114.5 mg/dL. Mean venous pH was 6.84 ± 0.03 with an anion gap of 30.3 ± 2.9 and a venous HCO_3_ level of 3.62 ± 1.35 mmol/L. Serum sodium and potassium levels were 134.7 ± 6.9 mg/dL and 4.77 ± 1.11 mg/dL, respectively. Mean calculated osmolality was 302.4 ± 12 mosm/L. All patients had acute renal failure on admission, with a mean serum creatinine of 1.57 ± 0.35 mg/dL compared to 0.55 ± 0.21 mg/dL at discharge. The rest of the routine biochemical variables can be found in Table 2.

### 3.4. Treatment

All patients received regular insulin infusion and aggressive fluid repletion. Mean total insulin infusion dose was 181.7 ± 90.4 U hours (on average 0.14 ± 0.05 U/Kg/h). Mean time on infusion was 24.4 ± 12.6. Initial potassium supplementation was needed (due to serum potassium on admission < 5.2 mg/dL) in 11 (38.8%) patients, and phosphorus supplementation was required only in a single patient. In total, 12 (66.6%) patients received bicarbonate infusion and all patients received prophylactic subcutaneous nonfractioned heparin (5,000 UI, BID). Upon discharge patients received education centered on management of diabetes and diabetes-related complications.

### 3.5. Clinical Course

We recorded no mortality in this case series. Mean in-hospital stay was 5.0 ± 4.1 days. The time from admission until resolution of acidosis (pH > 7.3 and HCO_3_ > 18 mm/L) was 16.6 ± 9.9 hours.

## 4. Discussion

In this study we describe the demographic, clinical, and biochemical characteristics associated with type 2 diabetes patients with very severe DKA. Several features we encountered, including signs, symptoms, and precipitating factors, were those classically described in standard cases of DKA. All patients responded adequately to treatment, and resolution of acidemia was accomplished within the first 24 hours in the majority of the patients. There was no in-hospital mortality.

The occurrence of DKA in type 2 diabetes is relatively unusual, but in the last decade several case series have explored its most relevant features. Between 13 and 21% of DKA admissions involve patients with type 2 diabetes [7–9]. The influence of diabetes type on severity of DKA is equivocal. In a series of 138 patients with DKA, type 1 diabetes patients were severely more acidotic, but type 2 diabetes patients required longer insulin infusion time [7]. However, another series of 208 patients reported a threefold increase of severe DKA in patients with type 2 diabetes compared with type 1 diabetes [8]. Other series have reported similar rates of severe DKA between diabetes types [9, 10]. Insulinopenia could be one of the main mechanisms driving the development of DKA in these cases [10].

Latin American heritage seems to be a predisposing risk factor for the development of DKA in type 2 diabetes patients [7, 11]. Pinto et al. reported a series of 53 Latin American patients with type 2 diabetes and DKA with a mean pH of 7.15 ± 0.14; bicarbonate of 7.73 ± 6 mEq/L; and anion gap of 24.45 ± 7.44 mEq/L [12]. In that series, over fifty percent of the cases were defined as severe forms of DKA, but mortality was zero, a finding in accordance with our observations. Discontinuation of medication use and infection were the most common precipitating factors of DKA, and newly diagnosed type 2 diabetes is not uncommon [7, 12]. When encountering a patient with DKA and type 2 diabetes, the presence of conditions such as latent autoimmune diabetes of adults and type 1.5 diabetes should be considered [13].

The lack of information about the use of bicarbonate therapy and patient's evolution with very severe acidemia (pH < 6.9) is widely recognized, and clinical trials in this scenario will be very difficult to complete [14]. After some studies involving large case series of patients with severe DKA, little differences were found in clinical outcomes when considering the degree of acidosis as an isolated variable [5]. The full and fast recovery as well as a null mortality that characterized our patients, regardless of whether or not they received bicarbonate therapy, provides additional support for the idea that the degree of acidosis does not determine the outcome of DKA in patients with type 2 diabetes. Indeed, in a large pediatric case series, it was observed that children with pH values as low as 6.73 promptly recovered, even without bicarbonate or additional therapy [6]. However, in a case series of insulin-dependent DKA in children from a developing country, severe acidosis was weakly correlated to mortality, but osmolality at admission was a better predictor of death [15]. In another series of adult patients with DKA, multiple organ failure scores, but not the level of acidemia, were found to correlate with mortality [16]. Our patients were all adults with non-insulin-dependent diabetes, and the different populations studied could explain these discrepancies. We suggest that the severity of acidemia may influence mortality in children with type 1 diabetes, but this does not appear to be the case in adults with type 2 diabetes.

The objective of therapy in DKA has historically placed importance on the rapid reversal of acidemia, following evidence that cerebral function in DKA is related to severity of acidosis, even without cerebral edema [14]. However, despite severe acidemia, only 4 of our patients presented altered state of consciousness and none developed seizures. There was also no evidence of cerebral edema in any of the patients, although it is known that this feared complication is much more common in children and adolescents [17].

Severe acidemia has also been associated with impaired hemodynamics, including peripheral vasodilation, decreased cardiac output, and refractory shock in animal studies [18, 19]. In children with type 1 diabetes, some case series have found that the severity of DKA is indeed associated with the presence of shock, and when shock is fluid refractory, this in turn correlates to cerebral edema and death [20, 21]. However, a recent systematic review revealed that there is no evidence of improved hemodynamic stability with the use of bicarbonate administration in severe DKA [14]. In fact, evidence of altered hemodynamics due to very severe acidemia in adult patients with isolated DKA comes only from single case report [22–24]. We did find that elevated levels of lactate on admission were common in our patients, but only two were found to be mildly hypotensive. These alterations resolved in all patients with fluid therapy, suggesting that fluid/hydration state and/or sepsis are more important to hemodynamics than the severity of acidemia. In the case of hemodynamics, these findings could support that there are differences in susceptibility between age groups (children versus adults) and diabetes type, as well.

## 5. Conclusion

Very severe DKA in type 2 diabetes is not uncommon in our population, shares many features with non-very-severe cases of DKA, and can be managed following standard published or institutional guidelines. Although our sample was small, we observed no mortality and favorable clinical courses. These types of observations undermine the motivation behind aggressive and controversial interventions such as bicarbonate therapy.

## Figures and Tables

**Table 1:** Patient clinical and demographic data.

	Patients (*n* = 18)
Female *n* (%)	11 (61.2)
Male *n* (%)	7 (38.8)
Age (years)	40.66 (23–59)
Time with DM diagnosis (years)	5.27 ± 3.12
Previous DKA	7 (38.8%)
BMI	22.9 ± 2.9
*Cause *	
Treatment omission *n* (%)	8 (44.4)
Infection *n* (%)	7 (38.8)
Other *n* (%)	3 (16.6)

BMI: body mass index was calculated by height meters^2^  × weight (Kg).

DKA: diabetic ketoacidosis; DM: diabetes mellitus.

**Table 2:** Laboratory and biochemical variables on admission.

Variable	Value
Glucose (mg/dL)	613.8 ± 114.5
pH	6.84 ± 0.03
HCO_3_ (mmol/L)	3.62 ± 1.35
AG	30.3 ± 2.9
Osmolality	302.4 ± 12
Potassium (mg/dL)	4.77 ± 1.11
Na (mg/dL)	134.7 ± 6.8
Cl (mg/dL)	93.3 ± 6.0
PO_4_ (mg/dL)	5.8 ± 2.15
Ca^+^ (mg/dL)	9.15 ± 0.86
Platelets (k)	445 ± 184.4
Creatinine	1.57 ± 0.35
WBC (k)	21.35 ± 5.0

HCO_3_: bicarbonate; AG: anion gap; RR: reference range; WBC: white blood cells; Hb: hemoglobin.

## Abbreviation

DKA: Diabetic ketoacidosis.
